# Correction: Photocatalytic degradation of drugs and dyes using a machine learning approach

**DOI:** 10.1039/d4ra90045f

**Published:** 2024-04-22

**Authors:** Ganesan Anandhi, M. Iyapparaja

**Affiliations:** a Department of Smart Computing, School of Computer Science Engineering and Information Systems, Vellore Institute of Technology Vellore 632014 Tamil Nadu India iyapparaja.m@vit.ac.in

## Abstract

Correction for ‘Photocatalytic degradation of drugs and dyes using a machine learning approach’ by Ganesan Anandhi *et al.*, *RSC Adv.*, 2024, **14**, 9003–9019, https://doi.org/10.1039/D4RA00711E.

The authors regret that the title shown in the original article was incorrect. The correct title is as shown above.

The Royal Society of Chemistry apologises for these errors and any consequent inconvenience to authors and readers.

## Supplementary Material

